# Hospitals in India

**Published:** 1893-12-16

**Authors:** 


### HOSPITALS IN INDIA.

### I.—THE BENGAL PRESIDENCY.

5. Civil Hospitals and Charitable Dispensaries of the Hyderabad Assigned Districts.

#### A.—Civil Hospitals.

At each district headquarter station, and at Chikalda and Kalamkhar, there is a civil hospital wholly supported by Government, and primarily intended to afford medical aid to police sick, and other Government servants; but admission is granted to the general public when there is available accommodation. The districts are six in number, viz., Akola, Basim, Buldana, Amraoti, Wun, and Ellichpur, near to which last are Chikalda and Kalamkhar, and the total population, according to the census of 1891, is 2,897,040 persons. The number of beds available was only 125, of which no more than 12 were for women. Indoor patients numbered 2,077 in 1891, and of these 1,844 were cured, 21 relieved, 108 discharged "otherwise," and 60 died, or 2·8 per cent. of the total treated. On an average only about half of the available accommodation was occupied daily, the actual figures being 61·11; but at Yeotmal and Ellichpur the daily average number of indoor patients was about equal to the number of beds, and in the sickly months in excess of it, so that it is to be hoped the accommodation at these institutions will be increased. Out-patients numbered 42,301, with a daily average of 211·44 of the total 32,911 attended personally, and 9,390, or 22 per cent., were represented by friends. The surgical work of these civil hospitals was represented by 153 major and 1,822 minor operations, as compared with 79 and 1,427 respectively in the previous year. Great importance is attached by the authorities to the surgical work, as successful operations tend to bring credit to the institution and induce the natives to apply for aid.

An abstract of the financial statements of the civil hospitals shows that the gross revenue amounted in 1891 to Rs.52,498 of which Rs.48,004 was paid by Government as salaries and special allowance. Rs.2,178 was provided from local funds, and Rs.2,298 was realised on European medicines. The total revenue was larger by Rs.2,196 than in 1890.

The total expenditure amounted to Rs.52,482, as against Rs.50,302 in the previous year. Of this total, Rs.42,536 was spent on establishment; Rs.4,239 on buildings or repairs; Rs.2,298 on European medicines supplied by Government and purchased locally; and Rs.2,449 on miscellaneous charges. Only Rs.611 was expended on diet, paupers being, as a rule, the only class dieted, although policemen are occasionally fed at Government expense, for when they are disabled from duty by their own imprudence their pay is disallowed by the police department, and, having no private means, they are obliged to be treated as hospital paupers, and dieted as such. The Inspector's comment on this arrangement deserves quotation: "It seems very absurd to punish a man in the police department by refusing him a sustenance allowance when in hospital, and then for the medical department to maintain him there till cured and able to rejoin the force. Either way Government pays the cost, and to debit it to the medical department is only robbing Peter to pay Paul, so far as the police department is concerned." The average cost of each diet ranges from 2 annas to 3 annas 9 pies.

#### B.—Charitable Dispensaries.

There were 36 of these institutions in the Hyderabad assigned districts at the end of 1891, of which 29 receive in-patients, and have an available accommodation of 163 beds. Taking into account the civil hospitals already alluded to, this gives 44 institutions where medical relief is obtainable, and 288 hospital beds for a population of 2,897,040 persons. Indoor patients at the dispensaries numbered 1,460, with a daily average attendance of 39·18, thus employing about a quarter of the available accommodation. The people still prefer outdoor relief, and it is chiefly sick travellers or wandering mendicants who apply for admission as indoor patients. Of the total number received, 78 per cent. are returned as cured, 5 per cent. as relieved, 5 per cent. as discharged "otherwise," and 10 per cent. died, as compared with 4·6 per cent. in the previous year. Of the 148 deaths reported, 26 died within twenty-four hours of admission, and the number of moribund cases admitted was very large. Out-patients numbered 236,913, of whom 198,691 attended personally and 38,222 were represented by friends. The daily average attendance was 1,592·53, and the average number of treatment days for each patient was 2·4.

The major surgical operations numbered 89, with 78 cures,

11 discharges (relieved or otherwise), and 4 deaths. The minor operations numbered 7,538.

The financial statements show that the invested cash balance at the beginning of the year was Rs.51,884 7a. 9p., and the floating balance was Rs.30,802 6a. 6p., a total balance of Rs.82,686 14a. 3p. The actual revenue during 1891 amounted to Rs.57,294 7a. 3p., of which Rs.28,650 was as salaries and special allowances from Government, Rs.10,284 came from local funds, Rs.7,655 from municipal funds, Rs.2,658 from interest on investments, Rs.4,000 from native subscriptions, and Rs.4,017 came as European medicines. European subscriptions amounted to Rs.23. The total balance and receipts were thus Rs.1,39,981 5a. 6p. The expenditure amounted to Rs.56,826 14a. 5p., of which Rs.34,422 went on establishment, Rs.10,614 on buildings and repairs, and Rs.5,320 on miscellaneous charges. None of the items call for special comment. The invested cash balance at the end of the year was Rs.54,984 7a. 9p., and the floating balance was Rs.28,169 15a. 4p., giving a total balance of Rs.83,154 7a. 1p.

Taken altogether, the civil hospitals and charitable dispensaries in these districts seem to make adequate provision to meet the demands of the population. A new dispensary is to be opened this year. The state of affairs with regard to subscriptions appears very unsatisfactory, and the Deputy Commissioner at Wun writes: "People are most unwilling to subscribe annually; they consider it is the business of Government to support the dispensaries, and that a donation once given should secure absolution to the donor from ever being asked to contribute again." Surely this view must be modified according to the amount of the donation, and in the meantime the Sanitary Commissioner suggests that where private subscriptions are not forthcoming annually to a certain amount the dispensaries should be closed. To do this would be to make a public and unfortunate confession of indifference and lack of charity, and would be a cause of shame to the district in which it occurred.

